# Exon level integration of proteomics and microarray data

**DOI:** 10.1186/1471-2105-9-118

**Published:** 2008-02-25

**Authors:** Danny A Bitton, Michał J Okoniewski, Yvonne Connolly, Crispin J Miller

**Affiliations:** 1 Cancer Research UK, Applied Computational Biology and Bioinformatics Group, Paterson Institute for Cancer Research, The University of Manchester, Christie Hospital Site, Wilmslow Road, Manchester, M20 4BX, UK; 2 Cancer Research UK, Proteomics Service, Paterson Institute for Cancer Research, The University of Manchester, Christie Hospital Site, Wilmslow Road, Manchester, M20 4BX, UK

## Abstract

**Background:**

Previous studies comparing quantitative proteomics and microarray data have generally found poor correspondence between the two. We hypothesised that this might in part be because the different assays were targeting different parts of the expressed genome and might therefore be subjected to confounding effects from processes such as alternative splicing.

**Results:**

Using a genome database as a platform for integration, we combined quantitative protein mass spectrometry with Affymetrix Exon array data at the level of individual exons. We found significantly higher degrees of correlation than have been previously observed (r = 0.808). The study was performed using cell lines in equilibrium in order to reduce a major potential source of biological variation, thus allowing the analysis to focus on the data integration methods in order to establish their performance.

**Conclusion:**

We conclude that part of the variation observed when integrating microarray and proteomics data may occur as a consequence both of the data analysis and of the high granularity to which studies have until recently been limited. The approach opens up the possibility for the first time of considering combined microarray and proteomics datasets at the level of individual exons and isoforms, important given the high proportion of alternative splicing observed in the human genome.

## Background

A number of studies have considered whether changes in mRNA concentration are reflected by similar changes in protein abundance e.g. [1-4]. Typically, poor correspondence has been reported between transcript and protein levels, and in some cases, little or no correlation at all e.g. [4]. Given the complexities of protein expression, and the many stages at which it may be influenced and/or actively controlled, this is perhaps to be expected.

Analogies can be drawn with studies that aim to compare microarray data generated using different platforms. These have highlighted the importance of filtering to remove data with poor signal to noise ratio, and the need to restrict comparisons to those pairs of reporters that target the same transcript location [5-7]. Transcript location is particularly important because approximately 50% of all human genes are predicted to be alternately spliced [5,8]. In many cases, different exons from the same gene will be represented in the spliced mRNA complement of a cell at different abundances. Most microarrays (e.g. the HGU133plus2 array from Affymetrix) typically offer only a single reporter per gene, and target locations differ between platforms. Cross mappings have generally been performed simply by seeking reporters annotated to the same gene identifier. The alternative, of restricting analyses only to those reporter-pairs that target the same transcript location, results in a significant proportion of the data being lost. A new generation of arrays have been developed with sufficient feature density to target every known and predicted exon in the human, mouse or rat genomes [5]. These 'exon' arrays offer a choice of probeset location when performing comparative studies, and allow cross-mapping to be performed with high precision, while still preserving much of the gene-level array data [5]. Numerous studies have also observed that differences in, for example, sample preparation, hybridization conditions and binding affinities can all have a significant influence on measured expression level. This makes direct comparisons between raw intensities challenging, although good correspondence is reported when comparisons are made using ratio data, e.g. [6].

We investigated whether similar considerations could be applied to quantitative mass spectrometry proteomics data generated using the iTRAQ reagent system [9]. iTRAQ (isobaric Tags for Relative and Absolute Quantitation, Applied Biosystems, ABI) is an extension of other approaches to protein identification (reviewed in [10]), in which a protein sample is fragmented and then separated into distinct peptides by liquid chromatography (LC). Peptides are then analysed using mass spectrometry to further fragment them and measure the mass to charge ratio for each resultant ion. *In silico *comparison of the resulting ion signature against a database generated from known protein sequences is then used to identify each peptide, and thus their originating proteins.

iTRAQ, like other similar approaches such as iCAT and SILAC [4], adds an additional labelling step following the initial fragmentation in which a chemical tag is attached to each peptide. The aim is to label every peptide in an experimental sample with a molecule of known mass (referred to here as 'the reporter group') and use the relative abundance of these reporter groups to determine the relative abundance of each peptide across samples. The mass spectrometry system is set up such that each (labelled) peptide is analysed separately. Multiple samples are compared in a single MS run, each labelled with a different sized reporter group. In addition, each tag contains a balance group, designed such that the labels used for each sample have the same overall mass. This has the consequence that all instances of a given peptide are processed at the same time by the mass spectrometer, irrespective of the experimental sample from which they originated, and has been shown to lead to improved accuracy [11,12]. When the labelled peptide is processed in the mass spectrometer it fragments, resulting in a series of ions corresponding to the fragmented peptide (which are used for identification in the usual way) and an additional set of peaks corresponding to the reporter tags for each sample. By determining the relative intensities of these reporter peaks, the relative abundance of a given peptide can be compared between samples.

Since the major focus of this paper is to consider whether the increased precision offered by exon arrays can be used in combination with proteomics data in order to improve the quality of the mappings between the two data sources, a biological system was chosen in which relatively high correlation between protein and transcript data might be expected, thus making it possible to establish a baseline level of correlation in a well-controlled system. In previous work using cell-lines in equilibrium, for example [2], relatively high correspondence (r = 0.59) has been reported. Here, two cell lines in steady state were also chosen. They provide not only a ready source of high quality RNA and protein (thus minimizing a significant source of technical variation), but also a system in dynamic equilibrium in which a major source of biological variation is removed. The aim was to generate a well-controlled dataset with which to investigate and evaluate different data-integration approaches.

## Results

Protein and mRNA samples were extracted from the human breast cancer cell line MCF7, and MCF10A, a non-tumourigenic human breast epithelial cell line. Material was processed according to manufacturers' recommendations (full protocols are available in the supplementary data). These cell lines have been used repeatedly in a variety of validation studies, e.g. [5,13-15], and the microarray data have been previously validated using real time PCR and by comparison between array types [5,13]. Microarray data were pre-processed using RMA [16] in BioConductor [17]; proteomics data using ProteinPilot and ProGroup (Applied Biosystems, Warrington, UK).

**Figure 1:**
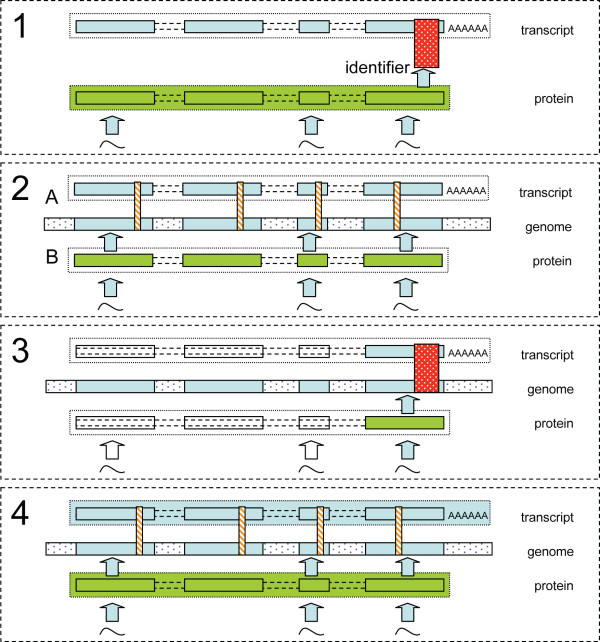
**Schematic of mapping strategies**. **(1) Conventional approach**. A summary value for each protein is mapped to its corresponding plus2 array probeset via SWISS-PROT sequence identifiers. **(2–4) Mappings performed via the genome**. **(A) **Exon array and plus2 array probesets are associated with their target exons using the X:Map database. These are then mapped to genomic coordinates via X:Map and Ensembl. **(B) **Peptide sequences are mapped to proteins using a sequence search against the Ensembl protein database and then mapped to genomic coordinates using the Ensembl Perl API. **(2) Exon array comparison**. Individual peptides are mapped to exons and then to their corresponding exon array probeset. **(3) Plus2 array comparison**. Individual peptides are mapped to exons and then to array probesets, as before. Since the majority of transcripts on a plus2 array are represented only by a single probeset placed at the 3' end of the transcript, many peptides cannot be mapped to an appropriate probeset. **(4) Exon array mappings**. A summary fold-change is created for the proteomics and microarray data by averaging across the peptides or probesets, respectively. These are then mapped via the genome to produce a comparison between transcript and protein level summaries.

In this section, mappings between the proteomics data and HGU133plus2 arrays and mappings between the proteomics data and Exon arrays are both considered. An overview of the approaches is shown in Figure 1.

### Transcript to protein comparison (plus2 arrays)

**Figure 2:**
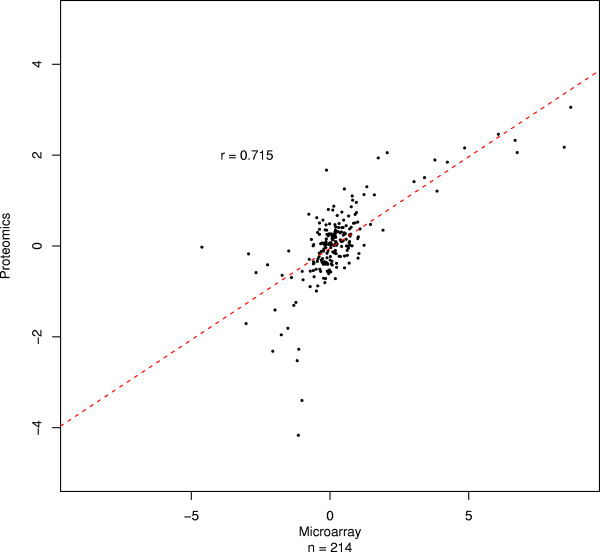
**High correspondence between plus2 array and quantitative proteomics data.** Each point corresponds to a matched transcript-protein pair using GeneCruiser annotation. X and Y axes: fold-changes between MCF7 and MCF10A for the microarray data and the quantitative proteomics data, respectively. When more than one matching probeset was found, the mean value was calculated, protein level averages were calculated by ProGroup. r: Pearson correlation. n: number of data points. No error bars are shown since these data are not provided by ProGroup.

In order to compare the method against current approaches, we first analysed the proteomics data alongside microarray data produced by hybridizing the RNA to HGU133plus2 arrays (Affymetrix, High Wycombe, UK). Protein level summaries were generated using ProteinPilot/ProGroup to search against the Celera database (Applied Biosystems, Warrington, UK). SWISS PROT identifiers, provided as part of the database annotation, were mapped to the array data using GeneCruiser [18]. 643 proteins were identified in this search (555 after filtering to remove low-quality data). 341 proteins were not mapped, either because they did not have an associated identifier (284/555 were found) or because no matching probeset was found (214/284 were found). In total, 214 proteins were mapped to 353 probesets, via 276 genes; figure 2. For these data, r = 0.715, p value of significance < 1 × 10^-16^. Similar results were observed when mapping via Genbank [19] accession and by searching other protein databases (data not shown).

This analysis represents the current state of the art, in which a single reporter (probeset) is used for the majority of transcripts, peptide data are summarised to provide a single value per protein, and mappings between the protein and the transcript data performed at the level of individual transcripts and proteins.

In order to investigate whether exon arrays could be used to enhance the quality of an integrated mRNA/proteomics analysis, a separate array study in which the same RNA was hybridized to Affymetrix Exon 1.0ST GeneChip^® ^(Affymetrix, High Wycombe, UK) arrays was also considered. Data were pre-processed using RMA, as before. High correspondence between these arrays and the HGU133plus2 arrays described above has been previously reported, demonstrating the intrinsic compatibility of both sets of array data [5].

### Probeset to peptide comparison (exon arrays)

Since exon arrays aim to provide a probeset in every known and predicted exon in the entire genome, they raise the possibility of performing a much more fine grained analysis than has been previously possible. This might be done by mapping individual peptides to the appropriate exon array probeset, using an annotation database to assign peptides to their originating exons, rather than by simply performing transcript and protein level summaries and mapping based on sequence id. To investigate this, a peptide-level analysis was conducted. A sequence search, combined with Ensembl [20], was first used to position peptides within their candidate proteins, after which they were aligned relative to the genome, and mapped to their originating exons using X:Map and exonmap [8]. X:Map and exonmap were then used to provided mappings to the exon array probesets. A peptide and probeset where mapped if they were found to originate from (or target) the same exon.

**Figure 3:**
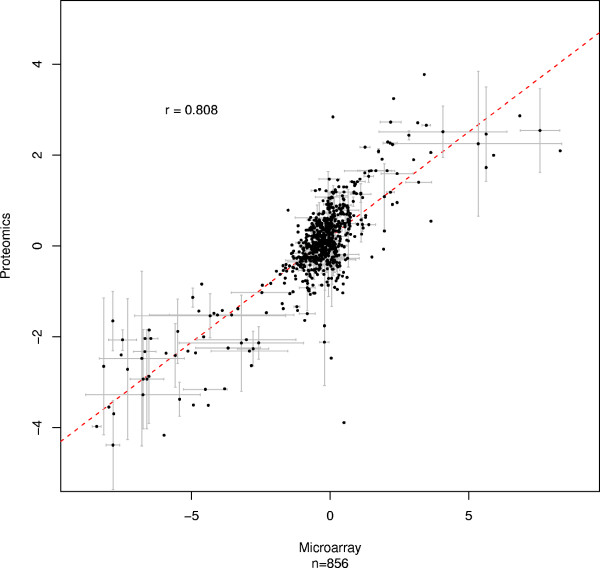
**High correspondence between exon array and quantitative peptide data.** Each point corresponds to an exon matched by at least one peptide and at least one array probeset. X and Y axes: fold-changes between MCF7 and MCF10A for the microarray probeset and quantitative peptide data, respectively. When more than one matching peptide/probeset was found, the mean value was calculated. Error bars represent one standard deviation. r: Pearson correlation. n: number of data points.

Another source of error in microarray studies arises because certain probes can hybridize to multiple targets [21]. An analogous situation can occur with peptides that match multiple places within the proteome. Data were thus filtered to remove probesets and peptides that may have originated from multiple locations in the genome [8]. From an initial set of 2,118 peptides, 1,934 remained after filtering for poor signal to noise ratio, and 1,168 after filtering for non-specificity. All peptides mapped to at least one exon, yielding 1,108 individual exons from 452 unique genes. When array mappings were performed, 856 of these exons mapped to 1,566 probesets, 1,094 of which were found by X:Map to target only a single location in the genome. Fold changes were computed for the remaining microarray and proteomics data, and their Pearson correlation found: r = 0.808 p < 1 × 10^-16 ^; figure 3. The difference in r (between figures 2 and 3) was also found to be statistically significant: p = 0.004, suggesting that exon arrays not only support mappings at the level of individual peptides but also lead to a marginal increase in the overall correspondence in the integrated dataset. Where multiple probesets (often corresponding to multiple start or end point predictions), and/or multiple peptides, are mapped to the same exon, these were averaged (error bars represent 1SD in these averaged values).

### Probeset to peptide comparison (plus2 arrays)

**Figure 4:**
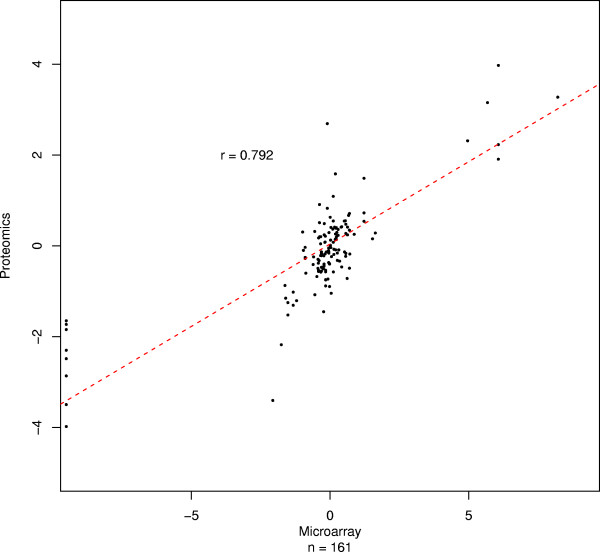
**High correspondence between plus2 array and quantitative proteomics data mapped by peptide proximity.** Each point corresponds to a matched probeset-peptide pair. A mapping was made when the genomic-origin of a peptide was within 1000 base pairs of the genome location of a microarray probeset. X and Y axes: fold-changes between MCF7 and MCF10A for the microarray data and the quantitative proteomics data, respectively. When more than one matching probeset was found for a given peptide, the mean value was calculated. r: Pearson correlation. n: number of data points. No error bars are shown because no averaging was performed because mapping was based on probeset/peptide location, rather than higher level features such as transcripts or exons, which provide a rationale strategy for grouping the peptides/probesets.

A similar analysis was performed using the HGU133plus2 data. Probesets were mapped if they matched the genome within 1,000 nucleotides up- or downstream of a peptide. Using this, less stringent mapping, only 161 peptides were successfully mapped to a corresponding HGU133plus2 probeset r = 0.792; p < 1 × 10^-16^; figure 4. An exon level mapping, as before, yielded only 91 successful peptide-matches, and as the size of the margin for an acceptable match increases, r decreases as expected (data not shown). These results demonstrate that although the approach is successful with plus2 array data it is only practical with high coverage arrays such as the Exon 1.0ST array; the relative paucity of probesets on the plus2 arrays means that very few are co-localized with a peptide, with the result that the majority of peptides are lost from the analysis.

### Transcript to protein comparison (exon arrays)

**Figure 5:**
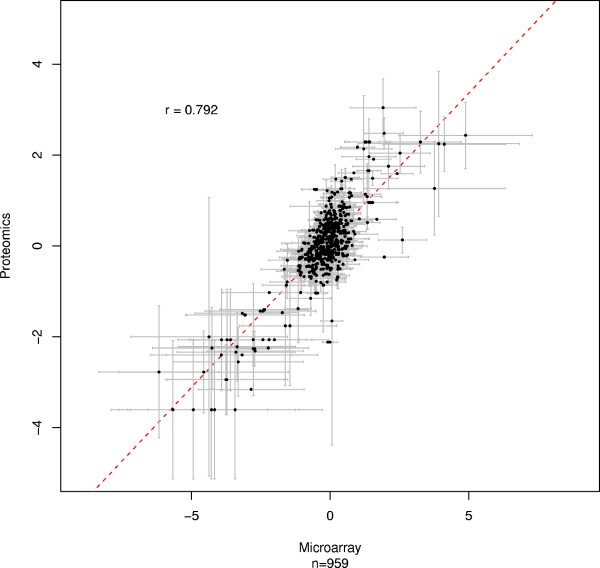
**High correspondence between protein and transcript level summary averages in exon array data.** Each point corresponds to a transcript matched by at least one peptide and at least one array probeset. X and Y axes: fold-changes between MCF7 and MCF10A for the microarray-probeset and quantitative-peptide data, respectively. When more than one matching peptide/probeset was found, the mean value was calculated. Error bars represent one standard deviation. r: Pearson correlation. n: number of data points.

We also considered the suitability of protein level summaries generated by averaging the individual fold-changes for all peptides matching to a given protein, as is commonly used to represent protein expression. We compared these values to their corresponding transcript level averages produced from the exon array data (figure 5; r = 0.792; p < 1 × 10^-16^). Although figure 5 shows high correlation between protein and transcript, many of the measurements also show high variance (the error bars in figure 4 represent 1 SD away from the mean). This is unlikely to be due simply to noise or technical artefacts, since there is highly significant correlation between individual probeset and peptide measurements, confirming their reliability (figure 3).

It is likely therefore, that much of this variation is a consequence of real biological events, such as alternative splicing. It is thus difficult to find a meaningful biological interpretation for protein or transcript level averages, since they mask a significant amount of underlying information that cannot be adequately represented as a single number. They should therefore be interpreted with care, even though correlation between them is high.

## Discussion

One of the major aims of this study was to perform a technically replicated experiment in which many sources of biological variation were controlled and thus to establish a baseline level of correlation between proteomics and microarray data. Thus the comparison was between two cell lines, allowing significant quantities of high quality RNA and protein to be produced, and, since each cell line was in a steady state, a major source of biological variation to be controlled [2,22]. This strategy is reflected in the high correspondence seen, not only in the HGU133plus2, but also the exon array comparisons, and is also a reflection of the quality of the data produced by both the iTRAQ and the Affymetrix systems. As mentioned in the introduction, previous work with steady state cell line data[2], has also reported substantial, albeit lower, correlation (r = 0.59), using different technology (ICAT and Agilent 60 mer arrays). Here we find significantly higher correlation (r = 0.808; difference in r; p = 9 × 10^-14^). Clearly, these results should be interpreted with some caution given the differences between the two studies, which include differences in the platforms used to perform the assays, the informatics approaches used to integrate the data and the biological system under investigation. However, the data presented here do show that high correlation between proteomics and microarray data can be observed in steady state cell line data.

In addition, exon arrays make it possible to show for the first time that it is feasible to use individual peptide ratios and compare them successfully to their corresponding exon array probeset by mapping via a genome annotation database. This is particularly important given the fact that at least half of the human genome is alternatively spliced. The substantial correlation found at the peptide-exon level, the high degree of variation observed within each individual data point at the transcript-protein level and the large amount of alternate splicing known to occur in the genome, together raise some substantial questions about the validity of averaged protein or transcript level summaries taken across the length of a 'gene'.

A criticism that can be made of quantitative proteomics approaches is that, in contrast to transcriptomics, it is not possible with current technology to fully characterize the entire proteome in a complex organism such as *homo sapiens*. However, the field is undergoing rapid development and it is likely that these issues will continue to be resolved as the technology progresses. The strategy described here relies on mapping reporters from the different platforms (i.e. peptides and microarray probesets) to one another via the genome. The approach is, therefore, relatively generic, and could equally be applied to other quantitative techniques.

## Conclusion

These findings suggest that much of the variation found between proteomics and microarray data occurs at least in part as a consequence of the data analysis techniques and the high granularity to which such studies have, until recently, been limited. When data processing takes into account the importance of alternative splicing, probeset location, issues of peptide/probeset selectivity and signal to noise ratio, a surprising degree of correlation is observed in the data. Where differences do remain, these are more likely to represent biological rather than technical effects, thus enhancing our ability to identify and pursue genes for which protein expression is modulated post-transcription.

## Methods

### Cell culture

MCF7 cells were grown in Dulbecco's modified Eagle's medium (DMEM) with 10% (v/v) fetal calf serum (FCS; Invitrogen, Carlsbad, CA, USA), and MCF10A was grown in DMEM/F12 with 5% (v/v) horse serum (Invitrogen, Paisley, UK), 2 ng/mL epidermal growth factor (PeproTech, Rocky Hill, NJ, USA), 0.5 μg/mL hydrocortisone, 0.5 μg/mL cholera toxin, and 5 μg/mL insulin (Sigma, St. Louis, MO, USA). Protein content per cell was similar for the two populations and all experiments were performed on sub-confluent cells cultured at the same density.

### Microarray processing

All experiments were performed using either Affymetrix HGU133 plus 2.0 or Exon 1.0ST arrays (Affymetrix, High Wycombe, UK). A complete description of procedures is available at [23].

• HGU133plus2 arrays

◦ GeneChip_Target_Prep_Protocol-CR-UK_v2.pdf

• Exon1.0ST arrays

◦ _HYBRIDISATION_PROTOCOLS/Exon 1.0 ST Hybridisation v1.0.pdf

Three technical replicates were performed for each microarray experiment.

### Protein preparation and iTRAQ labelling

Two million cells were washed with PBS, centrifuged at 500 × g for 5 minutes and the dried pellet lysed in 0.5 M triethylammonium bicarbonate + 0.05% (w/v) SDS. Protein was digested and iTRAQ labelled as described previously[24]. Briefly, 100 μg protein in 20 μl was reduced with 2 μl 50 mM tris-(2-carboxyethyl)-phosphine (TCEP) at 60°C for one hour and then alkylated with 1 μl of 200 mM methylmethanethiosulphate (MMTS) in isopropanol at room temperature for 10 minutes. Protein was digested by addition of 10 μl of trypsin at 0.5 μg/μl and incubated at 37°C overnight. One unit of iTRAQ reagent (Applied Biosystems, Warrington, UK), was thawed and reconstituted in 70 μl of ethanol, with vortexing for 1 minute. The reagent solution was added to the digest and incubated at room temperature for one hour. Labeling reactions were then pooled prior to analysis. Two technical replicates were performed. MCF7 cells were labelled with 114 and 116 reporter ions, MCF10A with 115 and 117.

### Liquid Chromatography and Mass Spectrometry

Pooled labelled peptides were analysed as previously described[24]. Briefly, peptides were fractionated on an SCX cartridge (Applied Biosystems) in 10 mM K2HPO4 (pH 2.7) + 20% ACN, with KCl concentration increasing in 50 mM steps from 50 mM to 500 mM. Peptide fractions were dried, and re-suspended in 240 μL 2% v/v ACN/0.1% v/v formic acid. 60 μL was loaded onto a 15 cm reverse phase C18 column (75 μm i.d.) using an LC Packings UltiMate™ pump and peptides separated on a 80 min gradient from 5% to 40% v/v ACN/0.1% v/v formic acid on-line to a QSTAR^® ^XL mass spectrometer.

### Data analysis

#### Relative Quantification and Peptide assignments

iTRAQ data analysis and peptide/protein database searches were performed using the ProteinPilotTM software (version 1.0, Applied Biosystems, Warrington, UK,). The uninterpreted spectra were searched against the human Celera protein database: human_KBMS5.0.20050302.fasta (187,748 proteins). Only peptide matches with a confidence >= 95% were considered. The program was configured to report methylmethanethiosulfate (MMTS) as a fixed modification.

### Microarray Data analysis

All analyses were performed using BioConductor/R[17]. Raw expression data were processed in R using the affy BioConductor libraries. Expression summarization was performed using RMA[16,25] with chip definitions supplied via a custom CDF file as described in[5]. Probeset to genome mappings were performed using exonmap[8].

### Peptide mappings

An overview of the data analysis strategy used to integrate the exon array and proteomics data is presented in figure 5. The set of distinct peptides reported by ProteinPilot (Applied Biosystems, Warrington, UK) following database search was extracted from the output file and filtered to retain only those peptides with >95% confidence, a contribution > 0 to the final protein identification, and flagged by ProteinPilot as being used for quantification). Then, the list was locally BLAST [26] searched (-M PAM30 -e 100 -W 2) against the human Ensembl [20] peptide database (Homo_sapiens.NCBI36.42.pep.all.fa), in order to retrieve Ensembl transcript IDs. This approach indirectly compares the Celera and the Ensembl databases. Minor discrepancies between the two databases therefore resulted in a small number of peptides not being mapped. The high e-value set for the BLAST search ensured that almost all possible hits were obtained. Nevertheless, only the exact peptide matches (i.e. 100% identity) of the same length as the query length were extracted. Finally, a BioPerl Ensembl API script [27] was used to pull out the peptides genomic coordinates. For peptides located within exon-exon junctions, two sets of coordinates were retrieved. Similarly, a peptide sequence that exists in more than one place in the genome (e.g. shared between protein families), would also have more than one set of coordinates. These 'multi-target' peptides were excluded from further analysis.

For comparisons involving the Exon1.0ST array data, peptides were mapped by location, using X:Map, to their 'parent' exons (figure 2) and transcripts (figure 4). Exonmap was then used to map these exons/transcripts to their corresponding probesets. Probesets that were found by X:Map to contain one or more probes capable of hybridizing to the genome at multiple locations were removed by filtering.

For peptide level comparisons involving the HGU133plus2 array data, plus2 probeset locations were found using ADAPT [28]. Peptides were mapped to a probeset either via exon, as before, or when one was found within 1000 residues of the peptide location.

### Protein to HGU133 plus2 comparisons

ProteinPilot was used to generate summaries, as before. These were then processed using ProGroup (Applied Biosystems, Warrington, UK) to generate protein level summaries. SWISS PROT identifiers, which form part of the Celera database annotation, were then mapped to probeset IDs using GeneCruiser [18].

### Correlation calculations

Pearson correlation was calculated as implemented by the 'cor' function in R and significance tested using t = r * sqrt(N-2)/sqrt(1-r^2^), where N is the length of the vectors and r is the correlation between them [29]. Significance of comparison between correlation coefficients was calculated using Fisher's transformation to z-scores. For exon level comparisons (figure 2), each data point corresponds to a peptide and probeset mapping to the same exon, and correlation calculated between the log_2 _fold changes reported for the peptides and probesets. Where multiple peptides or probesets mapped to the same exon, correlation was calculated using the mean peptide or probeset value for that exon. For these, error bars in the figure indicate one standard deviation away from the mean.

For transcript and protein level summaries using the exon array data (figure 4), the correlation is calculated between the mean log_2 _fold change for all exon targeting probesets and all peptides. As before, error bars in the figure indicate one standard deviation away from the mean.

For transcript and protein level summaries using the HGU133plus2 arrays, the correlation is calculated between the log_2 _fold change for the array probeset and the corresponding protein level average across peptides, determined using ProGroup, as described above.

## Authors' contributions

DB and MO performed the data analysis. YC performed the proteomics bench work. CM conceived the experiment and wrote the manuscript.

**Figure 6:**
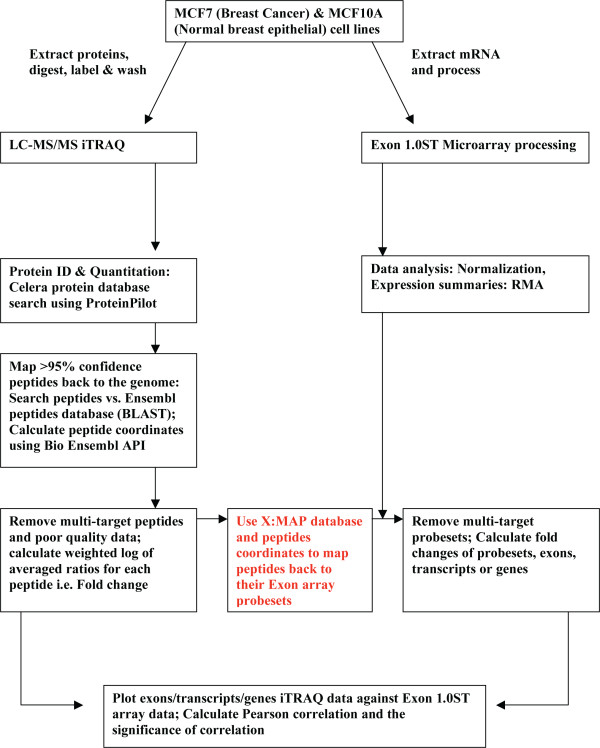
**Flow diagram showing the different steps required for the integration of iTRAQ and Exon 1.0ST data.** MCF7 & MCF10A cell lines were used as a source for protein and mRNA material. (LC) Liquid Chromatography; (MS/MS) Tandem Mass Spectrometry; (RMA) Robust Multichip Average.
